# Risk Factors of Acne Recurrence After Treatment and Establishment of an Early Warning Model

**DOI:** 10.1111/jocd.70545

**Published:** 2025-11-12

**Authors:** Keye Guo, Zhongming Lu, Huazhou Deng, Yan Shao

**Affiliations:** ^1^ Department of Dermatology Shengzhou People's Hospital, The First Affiliated Hospital of Zhejiang University, Shengzhou Branch Shaoxing China

**Keywords:** acne, early warning model, recurrence, risk factors

## Abstract

**Background:**

Acne vulgaris is a chronic inflammatory disease of the pilosebaceous units. It primarily manifests as comedones, papules, pustules, nodules, and cysts, making it the most common inflammatory skin disorder involving hair follicles and sebaceous glands. Acne predominantly occurs in adolescents and young adults. With continuous social development and increasing aesthetic standards, the demand for effective acne management has grown. However, as acne treatment is often prolonged and challenging, most patients experience recurrence after therapy, which significantly impacts their daily lives and emotional well‐being.

**Aim:**

This study aims to identify the factors associated with acne recurrence after treatment, and based on these findings, develop an early warning model to guide the prevention of acne recurrence.

**Methods:**

Clinical data from 218 patients were retrospectively analyzed. Among them, 105 cases (48.17%) experienced acne recurrence; 49 cases (46.67%) had mild recurrence; 37 cases (35.24%) had moderate recurrence; 19 cases (18.10%) had severe recurrence. Logistic regression analysis revealed that body mass index (BMI), smoking history, alcohol consumption, Self‐Rating Depression Scale (SDS) score, Self‐Rating Anxiety Scale (SAS) score, staying up late, poor eating habits, and prolonged use of electronic devices were risk factors for recurrence. In contrast, weekly pillowcase replacement was identified as a protective factor.

**Results:**

The predictive model was formulated as follows: 0.516 + (BMI × 0.283) + (smoking history × 0.775) + (drinking history × 0.854) + (SDS × 0.842) + (SAS × 0.744) + (staying up late × 0.396) + (eating habits × 0.935) + (time using electronic devices × 0.832) + (weekly pillowcase replacement × −0.512). The sensitivity, specificity, and AUC (95% CI) of the model were 88.57%, 80.53%, and 0.846 (0.791–0.891), respectively.

**Conclusion:**

The early warning model based on recurrence risk factors demonstrated strong predictive performance and may serve as a valuable tool in the clinical prevention of acne recurrence.

## 1 Introduction

Acne vulgaris is a chronic inflammatory disease of the pilosebaceous unit that predominantly affects the face, back, and chest of adolescents and young adults. Its clinical manifestations include comedones, papules, pustules, nodules, cysts, and scars [1]. Because facial acne is highly visible, it often contributes to depression, anxiety, and psychosocial distress [2]. The pathogenesis of acne involves multiple mechanisms, including androgen excess [3], massive cortical secretion [4], abnormal keratinization of cells around hair follicles [5], and inflammatory reaction [6]. Genetic susceptibility, psychological stress, and immune dysregulation may also contribute to disease onset or exacerbate its severity [7]. Current treatment strategies are tailored to pathogenic mechanisms and include reducing sebum production, inhibiting the proliferation of *Cutibacterium* acnes, regulating hormonal levels, and suppressing inflammation. Severe cases may require combined topical and systemic therapies administered over several months [8]. Although most patients present with mild to moderate disease, even mild acne can progress if inadequately managed. Therefore, in addition to treatment, reasonable disease management and family management are indispensable auxiliary methods [9].

Constructing an early warning model based on recurrence‐related risk factors may provide clinicians with an evidence‐based tool to improve preventive strategies. Accordingly, this study retrospectively analyzed the clinical data of 218 patients, including 105 with acne recurrence, to establish and validate an early warning model for predicting acne recurrence.

## 2 Baseline Characteristics and Methods

### 2.1 Baseline Characteristics

This was a retrospective cohort study. The clinical data and follow‐up information of all patients were collected from electronic medical records and archived questionnaire surveys at Shengzhou People's Hospital. A total of 218 patients who received acne treatment between January 2020 and December 2021 were included and categorized into two groups based on their recurrence status within 12 months after completing treatment: Recurrence group (*n* = 105, patients who experienced acne relapse); Control group (*n* = 113, patients who did not have recurrence).

Inclusion and exclusion criteria:

1. Inclusion criteria: Patients meeting the diagnostic criteria for acne vulgaris according to the clinical manifestations and grading system described in the 9th edition of Dermatology and Venereology (Zhang Xuejun editor) were enrolled. The diagnostic criteria required the presence of one or more of the following lesions: comedones (open/closed), inflammatory papules, pustules, nodules, cysts, or scarring. Disease severity was graded as: (i) Grade I (comedones only); (ii) Grade II (comedones with inflammatory papules); (iii) Grade III (comedones, inflammatory papules and pustules); or (iv) Grade IV (comedones, inflammatory papules, pustules with nodules/cysts/scarring). Additional requirements included: (v) voluntary treatment consent; (vi) ability to complete scheduled follow‐ups; and (vii) receipt of standardized treatment protocols under clinical supervision.
2. Exclusion criteria: Patients were excluded for any of the following: (i) severe primary diseases (cardiovascular, cerebrovascular, hepatic, renal or hematopoietic systems); (ii) psychiatric disorders; (iii) pregnancy/lactation; (iv) known hypersensitivity to study medications; (v) non‐compliance with treatment/follow‐up protocols; (vi) incomplete data documentation; or (vii) voluntary study withdrawal.

### 2.2 Ethical Approval

This study was approved by the Ethics Committee of Shengzhou People's Hospital (No. 2020‐K‐Y‐019‐01). The requirement for informed consent was waived due to the retrospective nature of the study. All data were de‐identified prior to analysis to protect patient privacy and confidentiality. This waiver is in accordance with the hospital's ethical guidelines and national regulations for retrospective studies involving de‐identified data.

## 3 Methods

### 3.1 Data Collection

Patients completed a questionnaire covering demographic and lifestyle information, including age, sex, family history of acne, sleep patterns, emotional status, dietary habits, and electronic device use. Staying up late was defined as sleeping after 11:00 p.m. on ≥ 3 nights per week. Dietary habits were categorized as frequency (e.g., spicy food ≥ 3 times/week).

### 3.2 Emotional Survey Scale

Anxiety was assessed using the Self‐Rating Anxiety Scale (SAS) scale. Patients rated 20 items, including forward‐scoring and reverse‐scoring items. The raw total score (*X*) was multiplied by 1.25 to yield the standard score (*Y*). Cutoffs: normal < 50, mild 50–59, moderate 60–69, severe ≥ 70.

Depression was assessed by the Self‐Rating Depression Scale (SDS). The SDS score was scored in the same way as SAS, but the cut‐off differs: normal < 53, mild 53–62, moderate 63–72, severe ≥ 73.

### 3.3 Assessment of Acne Severity

Acne was graded using the Pillsubury classification method: Mild: < 30 lesions, primarily comedones with few papules/pustules; Moderate: 30–50 lesions, moderate number of papules/pustules; Severe: > 50 lesions, numerous papules, pustules, nodules, or cysts.

### 3.4 Treatment Protocols

Patients received standardized therapy based on acne severity (Pillsbury classification):Grade I (Mild): Topical retinoid (0.025%–0.1% cream, nightly) + benzoyl peroxide (2.5% gel, daily); Grade II (Moderate): Topical combination + oral doxycycline (50 mg bid, 6 weeks); Grade III–IV (Severe): Oral isotretinoin (0.5 mg/kg/day, 16 weeks) or photodynamic therapy (weekly × 4); Treatment coding (*X*11): 1 = Topical monotherapy; 2 = Topical + systemic agents; 3 = Combined topical‐systemic‐physical therapy.

### 3.5 Follow‐Up

Recurrence was assessed through WeChat, email, telephone, and home visits. Outcomes were recorded and entered into a database.

### 3.6 Statistical Treatment

Data were analyzed using SPSS 22.0 (IBM Corp., Armonk, NY, USA). Continuous variables with normal distribution were expressed as mean ± standard deviation ($\bar{x}\pm s$) and compared using Student's *t*‐test. Categorical variables were presented as percentages (%) and compared using the chi‐square test. Logistic regression analysis was used to identify factors associated with recurrence. Predictive performance was evaluated with receiver operating characteristic (ROC) curve analysis. A *p*‐value < 0.05 was considered statistically significant.

## 4 Results

### 4.1 Investigation of Acne Recurrence and Severity

**FIGURE 1:**
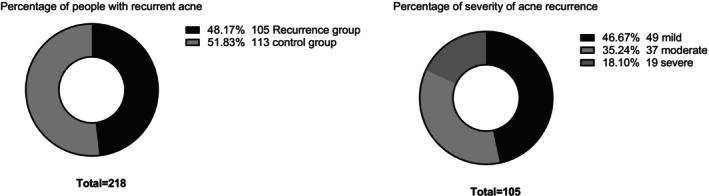
Proportion of patients with acne recurrence and proportion of severity.

At 12‐month follow‐up, acne recurrence and severity were investigated. Of the 218 patients, 105 cases (48.17%) had acne recurrence. Among them, 49 (46.67%) had mild recurrence, 37 cases (35.24%) had moderate recurrence, and 19 (18.10%) had severe recurrence (Figure 1).

### 4.2 Clinical Factors Associated With Acne Recurrence

Comparison of baseline characteristics between the recurrence group and the control group revealed significant differences in BMI, smoking history, drinking history, SDS, SAS, staying up late, family genetic history of acne, dietary habits, weekly times of makeup, time electronic device use, weekly pillowcase replacement, face washing frequency, and treatment modalities (Table 1).

**TABLE 1:** Clinical characteristics statistics.

	Recurrence group (*n* = 105)	Control group(*n* = 113)	(*χ* ^2^/*t*)/*p*
Age (years)	25.67 ± 5.53	25.31 ± 5.87	0.461/0.645
Gender			0.759/0.384
Man	58 (55.24)	69 (61.06)	
Woman	47 (44.76)	44 (38.94)	
BMI (kg/m^2^)	25.33 ± 1.49	21.33 ± 1.32	20.995/< 0.001
Smoking	61 (58.10)	43 (38.05)	8.763/0.003
SDS	66.06 ± 10.35	59.35 ± 9.14	5.076/< 0.001
SAS	63.81 ± 8.15	51.11 ± 9.76	11.062/< 0.001
Drinking	59 (56.19)	40 (35.40)	9.492/0.002
Staying up late	51 (48.57)	38 (33.63)	5.031/0.025
Family history of acne	55 (52.38)	36 (31.86)	9.426/0.002
Dietary habits			130.011/< 0.001
Spicy food	42 (40.00)	9 (7.96)	
Hot food	18 (17.14)	5 (4.42)	
Rich food	21 (20.00)	14 (12.39)	
Seafood	61 (58.10)	23 (20.35)	
Sweets	58 (55.24)	26 (23.01)	
Times of make‐up per week			4.496/0.034
≤ 2	38 (36.19)	57 (50.44)	
> 2	67 (63.81)	56 (49.56)	
Sun exposure per week			2.254/0.133
≤ 2	59 (56.19)	52 (46.02)	
> 2	46 (43.81)	61 (53.98)	
Electronic device use (h)	14.99 ± 3.32	11.19 ± 3.08	8.753/< 0.001
Weekly pillowcase replacement			7.909/0.005
Y	35 (33.33)	59 (52.21)	
N	70 (66.67)	54 (47.79)	
Face washing frequency			5.293/0.021
1–3	68	89	
≥ 4	37	24	
Treatment modalities			7.580/0.023
1	28	49	
2	52	48	
3	25	16	

### 4.3 Multivariate Analysis of Risk Factors for Acne Recurrence

Variables that differed significantly between the recurrence and control groups (Table 1) were included in the multivariate analysis. Face‐washing frequency (*X*10) and treatment modality (*X*11) showed strong multicollinearity with other covariates (variance inflation factor, VIF > 5) and were excluded to avoid model overfitting.

Multivariate logistic regression identified BMI, smoking history, drinking history, SDS, SAS, staying up late, dietary habits, and prolonged use of electronic devices as independent risk factors for acne recurrence. Weekly pillowcase replacement was an independent protective factor (Tables 2 and 3). The regression model demonstrated a good fit (*R* ^2^ = 0.295, *F* = 4.847, *p* < 0.05), indicating that at least one independent variable had a significant effect on recurrence.

**TABLE 2:** Variable assignment table.

Indicators	Variable	The assignment
Dietary habits	*X*1	1 = Spicy food, 2 = Hot food, 3 = Rich food, 4 = Seafood, 5 = Sweets
Weekly pillowcase replacement	*X*2	1 = Y, 2 = N
BMI	*X*3	—
Smoking	*X*4	1 = Y, 2 = N
Drinking	*X*5	1 = Y, 2 = N
Stay up late	*X*6	1 = Y, 2 = N
Electronic device use	*X*7	—
SAS	*X*8	—
SDS	*X*9	—
Face washing frequency	*X*10	1 = 1–3, 2 = ≥ 4
Treatment modalities	*X*11	1 = Topical monotherapy; 2 = Topical + systemic agents; 3 = Combined topical‐systemic‐physical therapy
Whether acne recurs	*Y*	1 = Y, 2 = N

**TABLE 3:** Logistic analysis of influencing factors of acne recurrence.

	Unstandardized coefficient	The standard error	The standard coefficient	*T*	*p*	VIF
Constant	2.842	0.382	—	4.738	< 0.001	—
Dietary habits	0.935	0.167	−0.765	−3.324	0.001	1.494
Weekly pillowcase replacement	−0.512	0.153	−0.432	−3.874	0.007	1.805
BMI	0.283	0.163	−0.874	−4.422	0.032	1.283
Smoking	0.775	0.123	−0.382	−3.786	0.043	1.673
Drinking	0.854	0.177	−0.697	3.968	0.003	1.482
Staying up late	0.396	0.144	−0.382	1.443	0.010	1.784
Electronic device use	0.832	0.135	−0.764	−1.234	0.009	1.382
SAS	0.744	0.178	−0.482	2.664	< 0.001	1.596
SDS	0.842	0.834	−0.582	−4.462	< 0.001	1.283
*F*			4.847			
*p*			< 0.001			
*R* ^2^			0.295			

*Note:* Dependent variable: acne recurrence.

**FIGURE 2:**
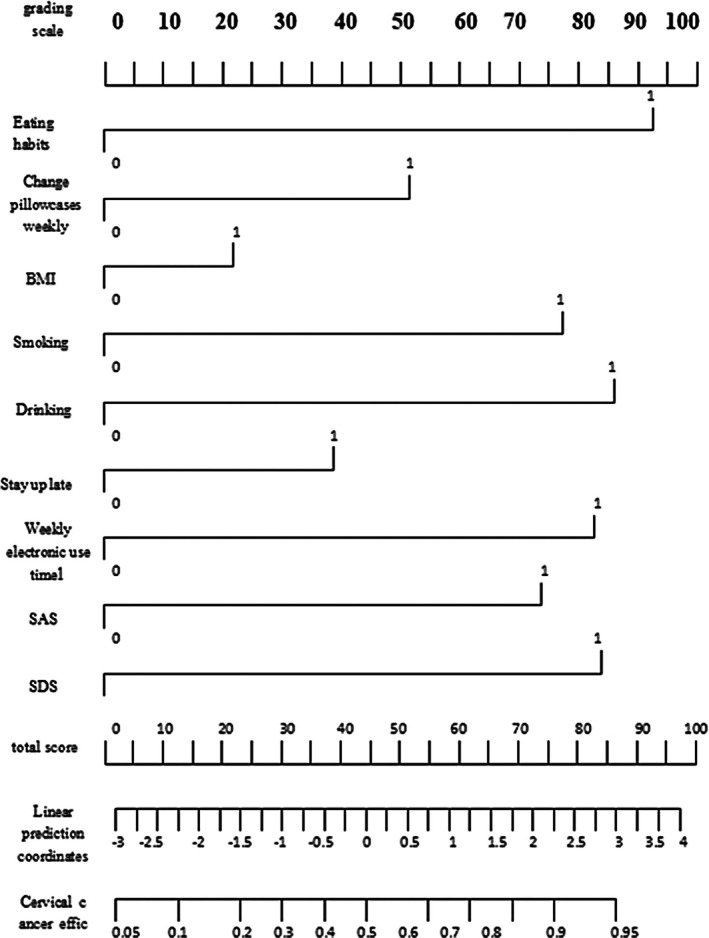
Nomogram of acne recurrence prediction model.

According to the above risk factors, a nomogram was drawn and a prediction model for acne recurrence was constructed. The model was as follows: Risk score = 0.516 + (BMI × 0.283) + (smoking × 0.775) + (drinking × 0.854) + (SDS × 0.842) + (SAS × 0.744) + (staying up late × 0.396) + (poor dietary habits × 0.935) + (electronic device use × 0.832) + (weekly pillowcase replacement × −0.512). Figure 2 is attached to the nomogram of the prediction model.

### 4.4 Validation of the Model for Acne Recurrence

**FIGURE 3:**
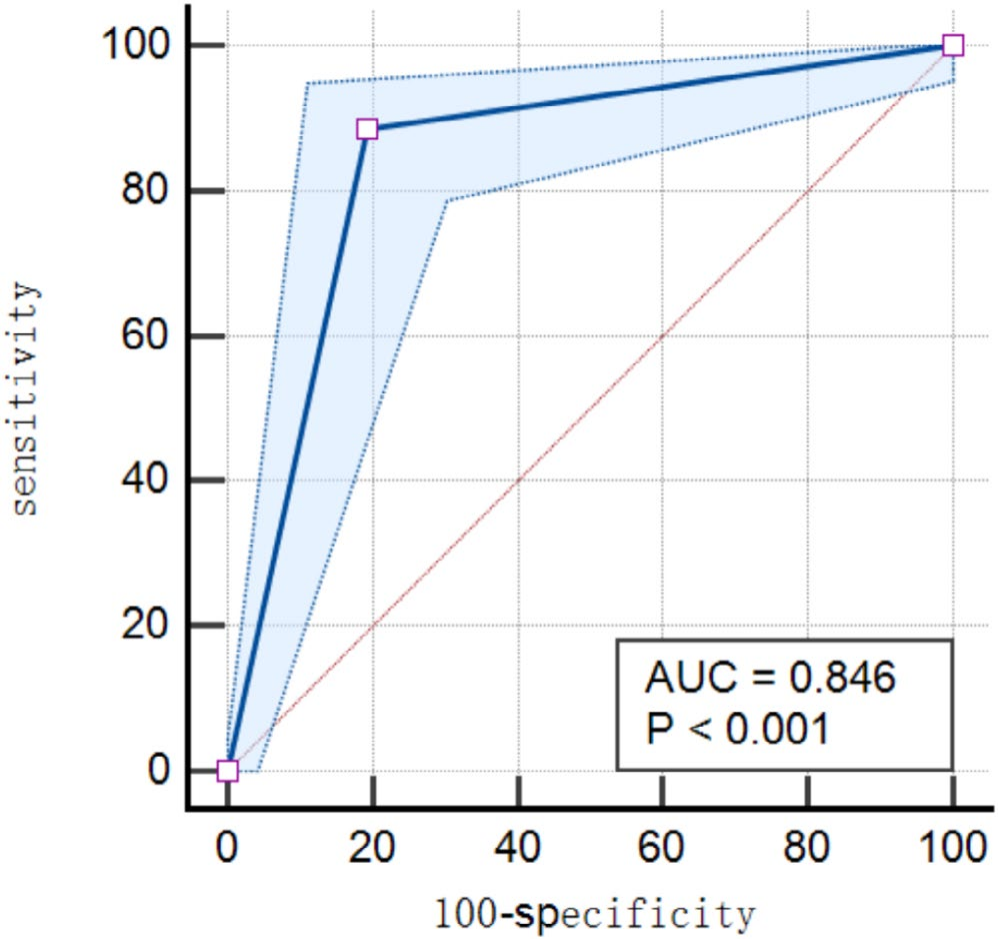
Predictive efficacy of the early warning model for acne recurrence.

ROC curve analysis was used to evaluate the model's predictive performance. A probability threshold of 50% was applied: patients with values ≥ 50% were classified as high risk for recurrence. The model demonstrated a sensitivity of 88.57%, a specificity of 80.53%, and an AUC of 0.846 (95% CI: 0.791–0.891) (Table 4, Figure 3).

**TABLE 4:** Predictive efficiency of early warning model for acne recurrence.

Acne warning model	Results follow‐up		Total
	Recurrence	No recurrence	
Recurrence	87	13	100
No recurrence	18	100	118
Total	105	113	218

## 5 Discussion

Sebum mixed with exfoliated epidermal tissue can form keratinous plugs that obstruct pilosebaceous ducts, initiating the development of acne [10]. In addition, bacterial colonization, abnormal follicular keratinization, sebaceous gland hyperactivity, and other pathogenic mechanisms contribute to acne onset [11]. Despite the availability of effective treatments for active acne vulgaris, recurrence following therapy remains a prevalent and distressing clinical outcome, contributing significantly to patient burden [12]. The findings of this retrospective cohort study elucidate that post‐treatment acne recurrence is not a random event but is significantly associated with modifiable lifestyle, psychological, and behavioral factors. Our multivariate analysis identified elevated BMI, a history of smoking and alcohol consumption, higher SDS and SAS, habitual late sleeping, unhealthy dietary habits, and prolonged electronic device use as independent risk factors. In contrast, weekly pillowcase replacement emerged as a protective factor.

The association between high BMI and acne recurrence reinforces the established link between obesity and a state of chronic, low‐grade inflammation. An elevated BMI fosters a pro‐inflammatory microenvironment, which can exacerbate vascular endothelial dysfunction and intensify cutaneous inflammatory responses, thereby creating a fertile ground for acne relapse [13]. Furthermore, the roles of smoking history and alcohol consumption as risk factors are supported by their known pro‐inflammatory properties. Nicotine from tobacco can aggravate systemic inflammation [14], while alcohol may induce facial flushing and stimulate the release of inflammatory mediators through catecholamine and bradykinin pathways, increasing the probability of recurrence [15].

Psychological distress also played a pivotal role. Elevated SDS and SAS scores were strongly associated with recurrence, consistent with literature documenting the bidirectional relationship between acne and psychological disorders [16]. The underlying mechanism may involve stress‐induced neurovascular and hormonal dysregulation, leading to the release of substance P and other neuropeptides that promote inflammation, vasodilation, and sebum production, thus facilitating relapse [17].

Our data also underscore dietary habits as a critical modifiable determinant. Patients in the recurrence group reported higher intake of spicy foods, seafood, and sweets. This supports previous studies linking high‐glycemic or pro‐inflammatory diets to endocrine disruption and androgen‐driven sebum overproduction, key drivers of acne pathogenesis [18].

An intriguing novel finding is the correlation between prolonged time spent using electronic products and recurrence. We hypothesize that the combined effects of light emission and heat from screens may potentially stimulate sebaceous gland activity. However, as the specific mechanistic pathway remains unquantified in current clinical literature, this association warrants further rigorous investigation.

The protective effect of frequent weekly pillowcase replacement is mechanistically sound. A clean sleeping surface minimizes facial exposure to accumulated sebum, bacterial colonies (e.g., Cutibacterium acnes), and microbial debris, thereby reducing the risk of follicular occlusion and inflammation that can lead to recurrence.

Although excluded from the final multivariate model due to collinearity, both face‐washing frequency (≥ 4 times/day) and treatment modality demonstrated significant univariate associations with recurrence, carrying important clinical implications. The higher rate of overwashing in the recurrence group supports the theory that aggressive cleansing can compromise the skin barrier integrity, increase transepidermal water loss, and provoke a rebound inflammatory response [19]. Regarding treatment, the observed lower recurrence rate in patients receiving combined physical therapy (e.g., phototherapy) aligns with international guidelines that endorse light‐based modalities as effective maintenance therapy to prevent relapse and improve long‐term outcomes [20]. Notably, a 2019 skin microbiome study suggested that our washing frequency variable may lack sensitivity [21], while the 12‐month follow‐up may not have been long enough to capture the sustained benefits of phototherapy recommended by European guidelines (2016). Therefore, future studies should adopt multicenter prospective designs, incorporate objective dermatological measures, and extend follow‐up to at least 24 months.

This study has several limitations. First, its retrospective design relies on the accuracy of medical records and self‐reported data, which may introduce recall bias. Second, the generalizability of our findings may be constrained by the single‐center nature of the study. Finally, the 12‐month follow‐up period may be insufficient to fully capture the long‐term preventive benefits of certain treatments; a longer follow‐up is recommended for future research.

In conclusion, acne recurrence is a multifactorial event profoundly influenced by lifestyle, behavioral, and psychological determinants. The early warning model developed in this study, which integrates these key risk factors, demonstrates strong predictive performance and offers clinicians a practical tool for identifying high‐risk patients and implementing personalized strategies to prevent relapse.

## Author Contributions

Keye Guo: conceptualization, formal analysis, visualization, writing – original draft. Zhongming Lu: methodology, software, validation. Huazhou Deng: methodology, supervision, writing – review and editing. Yan Shao: conceptualization, investigation, resources, writing – review and editing.

## Ethics Statement

This study was approved by the Ethics Committee of Shengzhou People's Hospital (No. 2020‐K‐Y‐019‐01). The requirement for informed consent was waived by the Ethics Committee due to the retrospective analysis of de‐identified clinical data, with all procedures complying with privacy protection regulations.

## Conflicts of Interest

The authors declare no conflicts of interest.

## Data Availability

The data used to support the findings of this study is available from the corresponding author upon request.
